# Genetic Mechanisms of Antimicrobial Non-Susceptibility to Novel Fluoroquinolone Delafloxacin Among Bulgarian Clinical Isolates of *Streptococcus agalactiae*

**DOI:** 10.3390/cimb47060446

**Published:** 2025-06-11

**Authors:** Vasil Boyanov, Alexandra Alexandrova, Raina Gergova

**Affiliations:** Department of Medical Microbiology, Medical Faculty, Medical University of Sofia, Zdrave Str. 2, 1431 Sofia, Bulgaria; v.boyanov@medfac.mu-sofia.bg (V.B.); alexandrova_sa@medfac.mu-sofia.bg (A.A.)

**Keywords:** delafloxacin, fluoroquinolones, *Streptococcus agalactiae*, GBS, antimicrobial resistance

## Abstract

Delafloxacin is one of the newest fluoroquinolones with a unique structure, determining better pharmacokinetic and pharmacodynamic properties, a better safety profile, and a broader spectrum of activity compared to older quinolones. We aimed to examine the susceptibility rates of delafloxacin, the genetic mechanisms contributing to resistance, and the serotype distribution in both invasive and non-invasive *Streptococcus agalactiae* strains. A total of 301 streptococcal strains were tested for minimal inhibitory concentration (MIC) to delafloxacin. All delafloxacin-resistant strains were subjected to serotyping, PCRs for quinolone-resistant genes, and sequence analysis for missense and silent mutations. Among the tested isolates, we found a 5.6% non-susceptibility rate to delafloxacin. The MICs ranged between 0.09 and 0.38 µg/mL, with a breakpoint for nonsusceptibility set as >0.03 µg/L, according to EUCAST criteria. All resistant isolates harboured missense mutations that led to amino acid substitutions in both GyrA (S81L) and ParC (S79F). Two common serotypes were determined among the resistant isolates: V (47.1%) and III (41.2%). Two strains were non-typable (11.7%). A statistical significance in the distribution of serotypes between delafloxacin-resistant and delafloxacin-susceptible strains was found. These findings highlight a concerning pattern of drug resistance developing prior to the introduction of a new medication, attributed to the extensive use of current antibiotics.

## 1. Introduction

Fluoroquinolones are broad-spectrum antimicrobial agents. They block bacterial topoisomerases type II-DNA gyrase and topoisomerase IV, responsible for the regulation of DNA supercoiling. This leads to disruption of the replication and transcription processes, DNA breakage, and activation of repair systems. When degradation processes prevail, the bacterial cell dies, which determines the bactericidal activity of this group of drugs [1].

The quinolone class includes compounds with a bicyclic ring chemical structure, divided into four generations. The first-generation representative is nalidixic acid. Subsequent generations were developed by adding a fluoride atom and additional modifications, yielding fluoroquinolones with improved pharmacokinetic parameters and an extended spectrum of activity [2].

Clinical studies indicate very good tolerance and a better safety profile for delafloxacin than most quinolones [3]. Some dose-dependent side effects such as peripheral neuropathies, allergic reactions, and *C. difficile*-associated diarrhoea have been reported, but are milder than other fluoroquinolones. Rare side effects such as hyperglycemic episodes and severe elevations of liver transaminases have also been described. Photosensitivity and cardiotoxicity have not been identified in studies to date. Like other quinolones, delafloxacin is not recommended during pregnancy, despite the lack of teratogenic effects in animal models, and is not advised for use in childhood [4,5,6].

Unlike all other fluoroquinolones with neutral amphoteric molecules (zwitterions), delafloxacin is the only one with anionic properties. This unique advantage helps the transport and accumulation of the antibiotic in the bacterial cell and phagolysosomes. It exhibits an enhanced antibacterial effect at acidic pH in inflammatory foci and biofilms. An additional chloride atom and an increased surface area of the molecule lead to enhanced activity against anaerobes and bacterial isolates resistant to other quinolones, respectively. It determines a broader spectrum of activity of delafloxacin [4,7]. It is classified as a fourth-generation fluoroquinolone and is approved by the Food and Drug Administration (FDA 2017) and European Medicines Agency (EMA 2019). It exhibits activity against numerous Gram (+) and Gram (−) bacteria, including methicillin-resistant *Staphylococcus aureus*, extended-spectrum beta-lactamase producing bacteria such as *Escherichia coli*, *Klebsiella pneumoniae*, as well as multidrug-resistant (MDR) *Pseudomonas aeruginosa*, *Helicobacter pylori,* and anaerobes (*Bacteroides fragilis*), as well as causative agents of atypical pneumonia (*Chlamydia pneumoniae*, *Mycoplasma pneumoniae*, *Legionella pneumophila*) [5,8,9,10,11,12].

The primary resistance mechanism is mutations in the quinolone resistance-determining regions of genes encoding topoisomerases (*gyrA* and *gyrB* for DNA gyrase; *parC* and *parE* for topoisomerase IV). Significant missense mutations leading to amino acid substitutions and quinolone resistance are mainly described in GyrA (S81L/Y/F; E85K/A) and ParC (S79F/Y/A; S80P; D83Y/N) [13,14,15,16,17,18]. Isolated mutations in *gyrB* and *parE* were found in norfloxacin and ciprofloxacin-resistant strains, with uncertain evidence of whether they have important roles in newer generations of fluoroquionolones [15,19,20]. Additional mechanisms include increased efflux and enzymatic modification by plasmid-encoded enzymes are rare [21,22]. Delafloxacin is unique among quinolones because it is equally effective against DNA gyrase and topoisomerase IV. This characteristic is believed to decrease the selection of mutant strains in vitro and in vivo [23]. Resistance to delafloxacin requires multiple point mutations in both topoisomerases, unlike most fluoroquinolones, where a single nucleotide substitution in one of the enzymes can result in non-susceptibility. Consequently, strains resistant to other quinolones are usually susceptible to delafloxacin in most cases [24,25,26]. However, isolated cases of delafloxacin-resistant Group B Streptococcus (GBS) have been reported [23].

Infections caused by GBS are among the approved indications of this antibiotic [5]. *Streptococcus agalactiae* (GBS) is an opportunistic pathogen and can cause invasive infections with various localizations [27,28,29]. GBS is a leading cause of neonatal meningitis and sepsis [30]. Immunocompromised patients and elderly people with chronic non-infectious diseases are mainly affected [31]. GBS can cause urinary tract infections, bacteremia, osteomyelitis, septic arthritis, meningitis, endocarditis, pneumonia, and skin and soft tissue infections [32,33]. In terms of skin and underlying tissue involvement, GBS may cause cellulitis, abscesses, diabetic foot infections, and decubital ulcers, as well as rarely necrotizing fasciitis and pyomyositis [31,34]. This pathogen has ten serotypes (Ia, Ib, II–IX), which are categorised based on their capsular polysaccharide antigens. The use of delafloxacin is currently limited to the treatment of skin infections and community-acquired pneumonia.

In our study, we investigated the susceptibility rates of delafloxacin, the genetic mechanisms that contribute to resistance against this antimicrobial agent, and the serotype distribution in both invasive and non-invasive *S. agalactiae* strains.

## 2. Materials and Methods

### 2.1. Specimen Collection

During routine diagnostics, we isolated 301 strains (n = 301) of GBS from outpatients and inpatients aged between 17 and 88 years. The samples were collected from three hospitals in Bulgaria between September 2021 to January 2025. Among them, 17 (n = 17) were resistant to delafloxacin. We categorised the samples into two groups according to their source. The first group (Group 1) consists of vaginal samples (n = 10) collected from pregnant and non-pregnant women with confirmed genital infection with GBS as either a co-infectious agent in bacterial vaginosis or as a leading pathogen in aerobic vaginitis. The second group (Group 2) contains 7 extravaginal samples divided according to their source: invasive materials (n = 4) collected from sterile sites such as blood culture (n = 1), soft tissue wound aspirates (n = 2), tracheal aspirates (n = 1), and non-invasive samples (n = 3): urine (n = 2) and ejaculate (n = 1).

### 2.2. GBS Strains

The methods for initial identification of GBS were Gram-stain, positive CAMP test, negative catalase, and PYR tests, no susceptibility to bacitracin, and positive latex agglutination for serogroup B by Lancefield (PathoDxtra Strep Grouping Kit ThermoScintific, Oxoid, Hampshire, UK). If necessary, subsequent biochemical identification was performed with Crystal GP (Beckton Dickinson, Kelberg, Germany).

*S. agalactiae* strains were stored at −70 °C and subcultivated three times on Columbia agar (Becton Dickinson, Kelberg, Germany) supplemented with 5.0% sheep blood for 20–24 h at 35 °C in 5.0% CO_2_ before use for antibiotic susceptibility testing and other tests. Reference strains *Streptococcus pneumoniae* ATCC 49619 and *S. agalactiae* ATCC 27956 were used to control the tests performed according to EUCAST guidelines (2025) [35].

### 2.3. DNA Extraction

DNA extractions from pure cultures of *S. agalactiae* were performed with the DNA-Sorb-A DNA extraction kit (Sacace Biotechnologies Srl, Como, Italy) according to the manufacturer’s instructions. All DNA extracts were stored at −70 °C.

### 2.4. Antimicrobial Susceptibility Testing

Antibiotic susceptibility testing to delafloxacin was performed by determining the minimum inhibitory concentration (MIC) using E-tests (Liofilchem, Roseti degli Abruzzi, Italy). For the interpretation of the results, we used the EUCAST criteria (EUCAST breakpoints, 2025 [35]), according to which at MIC values > 0.03 µg/L, the tested strains were considered resistant [35].

### 2.5. PCR Amplification of Quinolone-Resistant Genes

GBS identification, detection of capsule serotypes, and topoisomerase genes amplification were carried out by PCR using primers previously described [36,37,38]. For the conventional PCR reaction, we used prime Taq premix 2× (Genetbio Co., Ltd., Daejeon, Republic of Korea) and the result was read by gel electrophoresis using GelRed^®^ Nucleic Acid Gel Stain (Biotium, Inc., Fremont, CA, USA).

### 2.6. Automated Sequencing

The *gyrA* and *parC* PCR products were purified using Rapid PCR Cleanup Enzyme Set (ExoSAP, Applied Biosystems, Cleveland, OH, USA). Sanger sequencing was performed by BigDye^®^ Terminator v3.1 Cycle Sequencing Kit and BigDye^®^ Terminator v1.1 and v3.1 5× Sequencing Buffer, on Applied Biosystems 3130xl Genetic Analyzer. The obtained sequences were analysed and compared to *S. agalactiae* GTC 1234 (susceptible to quinolones strain, [14]) and *S. agalactiae* GTC 1966 (resistant to quinolones strain, [14]) using the Basic Local Alignment Search Tool (BLAST, v. 2.16.0) available at the National Center for Biotechnology Information (NCBI) (http://www.ncbi.nlm.nih.gov/BLAST, accessed on 26 June 2024).

### 2.7. Statistical Analysis

Statistical analyses were performed using IBM SPSS Statistics for Windows v19.0 (IBM Corp., Chicago, IL, USA). Fisher’s exact test was used. A *p*-value ≤ 0.05 was considered statistically significant.

## 3. Results

**Figure 1:**
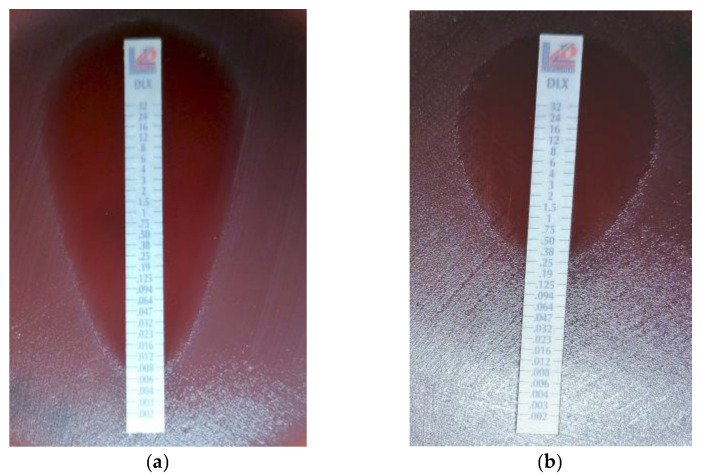
Determination of MIC by E-test (Liofilchem, Roseti degli Abruzzi, Italy). (**a**) Delafloxacin-susceptible GBS isolate; (**b**) delafloxaci- resistant GBS isolate.

Among the studied 301 GBS strains, the *S. agalactiae* isolates resistant to delafloxacin (n = 17) were 5.6%. According to the MIC values determined by E-test, the delafloxacin-resistant strains were grouped in a range between 0.09 and 0.38 µg/mL (Figure 1). Only one of the resistant strains showed a high level of non-susceptibility in the indicated interval, while most were determined with values around 0.19 µg/mL.

**Figure 2:**
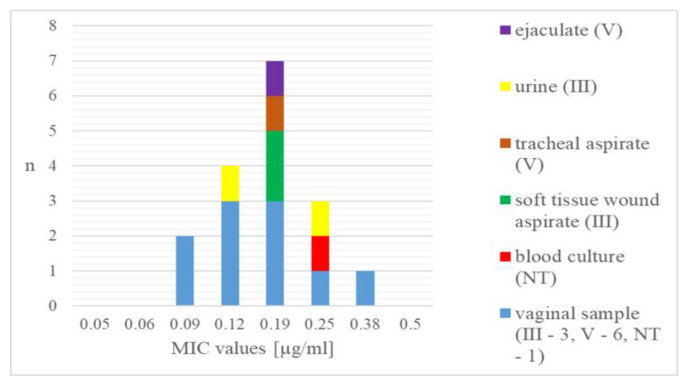
Distribution of delafloxacin-resistant GBS strains according to MIC values, specimens, and serotypes. n—number of strains; III and V—GBS serotypes; NT—non-typeable.

Regarding the type of specimen from which GBS was isolated, vaginal samples were represented in all examined MICs, while the other invasive and non-invasive isolates were mainly between 0.19 and 0.25 µg/mL (Figure 2).

The resistant isolates belonged to either serogroup III (41.2%) or V (47.1%), and two of them were non-typeable (11.8%) (Figure 2). There was statistical significance between the distribution of the serotypes in delafloxacin-resistant and delafloxacin-susceptible strains obtained with Fisher’s exact test (*p* < 0.05) (Table S1).

All tested strains (100%) harboured the same missense mutations in both *gyrA* (S79F, TCC → TTC) and *parC* (S81L; TCA → TTA). In addition, one of the isolates showed two more mutations with amino acid substitution in the *gyrA* N51D (AAT → GAT) and E52A (GAA → GCA) compared to GBS reference strains (GTC 1234 and GTC 1966) using BLAST available at NCBI (http://www.ncbi.nlm.nih.gov/BLAST, accessed on 26 June 2024). Silent mutations were detected in all examined isolates (Table 1).

## 4. Discussion

Delafloxacin is one of the newest fluoroquinolones included in the treatment regimens of problematic multidrug-resistant bacterial species. Although it is not yet widely used, resistance has been established in isolates collected from clinical samples. This resistance is primarily due to mutations in genes (*gyrA* and *parC*), leading to amino acid substitutions in both topoisomerases [4]. Most fluoroquinolones vary in their mechanisms of action against these two enzymes, and a single mutation can lead to an 8- to 16-fold increase in non-susceptibility, while double mutations are responsible for complete resistance [39].

In our study, all resistant isolates had significant missense mutations in both *gyrA* and *parC* genes, which were demonstrated to cause fluoroquinolone resistance [14,15]. This finding is in accordance with the previously reported data, which indicated that strains exhibiting no mutations or containing a mutation in one of the tested genes demonstrated susceptibility to delafloxacin [5,8]. We detected two additional missense mutations in *gyrA* in one of the isolates (N51D and E52A). Since no data supports their significance, they remain a subject for further investigation.

Investigation on 225 GBS strains (150 strains isolated in the USA and 75 in Europe) revealed 2.7% resistance to delafloxacin, with MIC values being as follows: 0.06 µg/mL^−1^, 0.12 µg/mL^−2^, 0.25 µg/mL^−2^, and 0.5 µg/mL^−1^ [23]. In comparison, we found more than twice higher resistance rates in GBS—5.6% of 301 isolates. Most of these resistant isolates had a MIC value of around 0.19 µg/mL, and the highest disclosed MIC level was 0.38 µg/mL. In contrast, the presence of mutations in both topoisomerase enzymes results in MIC greater than 32 μg/mL for levofloxacin. This highlights that microorganisms tend to have higher MIC values for other fluoroquinolones compared to delafloxacin [18,26,39].

Serotypes III, V, and Ia are predominantly associated with invasive infections, and serotype III is considered the most virulent [27]. In the current work, among all tested strains (n = 301), serotype Ia was predominant, while serotypes III and V made up a total of 40.5% (Table S1). In contrast, all identified delafloxacin-resistant strains were serotypes III or V, with a statistical significance of their distribution compared to susceptible ones. This highlights the selection of the most problematic serotypes as resistant to one of the newest fluoroquinolones.

In comparison to delafloxacin resistance, GBS exhibits a higher non-susceptibility to other antibiotics. In our research conducted from 2021 to 2024, GBS resistance rates to macrolides, lincosamides, and tetracyclines were found to be 60.3%, 24.9%, and 89.1%, respectively. However, all tested isolates were susceptible to both penicillin and vancomycin [40]. Regarding the fluoroquinolone resistance observed in another Bulgarian study, a 10.3% non-susceptibility to levofloxacin was identified in GBS, particularly in urine isolates [41]. In comparison, other countries observed a higher rate of levofloxacin resistance. In Turkey, the resistance was reported to be 27.6%, whereas in China, it varied between 10.3% and 72.9% [42,43]. The data presented highlighted the spread of MDR GBS and the significance of developing and testing new medications. Delafloxacin is recognised for its superior properties compared to older fluoroquinolones; however, it is important to assess antibiotic susceptibility prior to use due to the presence of accumulated mutations in bacterial populations.

The first approved use for delafloxacin is in the treatment of skin and soft tissue infections, including cellulitis, wound infections, and abscesses. When comparing the efficacy of delafloxacin monotherapy and the vancomycin/aztreonam combination for these infections, the two treatment regimens demonstrated very similar efficacy, where delafloxacin having a better safety profile. Another approved indication is community-acquired pneumonia [10,44,45]. Concerning other pathogens belonging to the *Streptococcaceae* family, delafloxacin is demonstrated to have a high activity against *Streptococcus pyogenes* and *Streptococcus dysgalactiae* [23]. According to EUCAST, there is insufficient evidence to support the use of delafloxacin for *Streptococcus pneumoniae* infections; however, research involving levofloxacin and cefotaxime-resistant *S. pneumoniae* demonstrated promising results [23,35,46,47]. This signifies that delafloxacin is a good option for the treatment of streptococcal infections. However, in the present study, GBS resistance was reported in two strains from wound aspirates and one from tracheal aspirate, cases in which empiric treatment with delafloxacin would be appropriate.

Although delafloxacin is still not used in daily practice in our country, we have observed the presence of strains with target modification resulting from accumulated mutations. This raises concerns about the emergence of MDR GBS isolates and questions the continued effectiveness of delafloxacin, especially as a monotherapy for treating uroinfections, skin, subcutaneous, and other infections.

## 5. Conclusions

Delafloxacin exhibits strong activity against *S. agalactiae* (GBS). However, despite its limited use in clinical practice in Bulgaria, we have identified 5.6% of non-susceptible strains. This indicates that there are a considerable number of strains with accumulated mutations in both genes that encode topoisomerase enzymes. The emergence of these resistant pathogens is linked to the widespread use of existing fluoroquinolones. Furthermore, these isolates were limited to serotypes that are typically linked to invasive diseases. These results highlight a worrying trend for developing resistance to new drugs even before their introduction.

## Figures and Tables

**Table 1:** Missense and silent mutations in the *parC* and *gyrB* genes.

	Missense Mutations		Silent Mutations	
	Substitution	Frequency	Substitution	Frequency
*parC*	S79F (TCC →TTC)	100%	A142A (GCG → GCA/GCC)	94.1%
			I81I (ATC → ATT)	5.9%
*gyrA*	S81L (TCA → TTA)	100%	G49G (GGT → GGG)	5.9%
	E52A (GAA → GCA)	5.9%		
	N51D (AAT → GAT)	5.9%		

S—serine; F—phenylalanine; A—alanine; I—isoleucine; L—leucine; E—glutamic acid; N—asparagine; D—aspartic acid.

## Data Availability

All datasets generated or analysed during the study are included in the manuscript.

## Supplementary Materials

The following supporting information can be downloaded at https://www.mdpi.com/article/10.3390/cimb47060446/s1.
