# Supplementary material for: Genetic Mechanisms of Antimicrobial Non-Susceptibility to Novel Fluoroquinolone Delafloxacin Among Bulgarian Clinical Isolates of Streptococcus agalactiae
Source: Curr Issues Mol Biol. 2025 Jun 11;47(6):446. doi: 10.3390/cimb47060446 (PMC12191815; doi:10.3390/cimb47060446)
Supplement: Supplementary file 1 [file cimb-47-00446-s001.zip › cimb-3640375-supplementary.pdf]

Table S1: Distribution of serotypes among delafloxacin resistant and delafloxacin susceptible GBS isolates.

Serotypes	Delafloxacin resistant (n=17)	Delafloxacin susceptible (n=284)	Total number (n=301)	p-value* (delafloxacin resistant/delafloxacin susceptible)
Ia	0	75 (26.4%)	75 (24.9%)	
Ib	0	4 (1.4%)	4 (1.3%)	
II	0	46 (16.2%)	46 (15.3%)	
III	7 (41.2%)	51 (18.0%)	58 (19.3%)	**0.027**
IV	0	24 (8.5%)	24 (8.0%)	
V	8 (47.1%)	56 (19.7%)	64 (21.3%)	**0.013**
VI	0	1 (0.3%)	1 (0.3%)	
VII	0	1 (0.3%)	1 (0.3%)	
VIII	0	0	0	
IX	0	0	0	
NT**	2 (11.7%)	26 (9.2%)	28 (9.3%)	0.664

* a p-value <0.05 is considered statistically significant.

**NT – non-typable.
